# Triglyceride-glucose index as a predictor of cardiovascular multimorbidity: a prospective cohort study

**DOI:** 10.3389/fcvm.2025.1647444

**Published:** 2025-11-14

**Authors:** Yushi Bao, Hao Chen, Bolin Zhu, Minhua Tang, Qiang Wu, Yingnan Jia, Yonggen Jiang

**Affiliations:** 1 Department of Clinical Labortatory, Shanghai Songjiang District Sijing Hospital, Shanghai, China; 2 Department of Preventive Medicine and Health Education, School of Public Health, Fudan University, Shanghai, China; 3 Department of Preventive Health Care, Zhongshan Community Health Care Center, Shanghai, China; 4 Songjiang District Center for Disease Control and Prevention, Songjiang District Health Inspecting Agency, Shanghai, China

**Keywords:** triglyceride-glucose index, cardiovascular multimorbidity, multimorbidity, prospective cohort study, cohort study

## Abstract

**Background:**

The triglyceride-glucose (TyG) index is a recognized marker of insulin resistance, yet its role in the dynamic progression from cardiovascular disease (CVD) to cardiovascular multimorbidity (CVM) remains unclear. This study aimed to evaluate the association between the TyG index and transitions from a CVD-free state to first CVD and subsequently to CVM.

**Methods:**

In this prospective cohort study, we included participants from the Shanghai Suburban Adult Cohort who were free of CVD at baseline (2016). The TyG index was measured at baseline. Disease transitions were tracked over a median follow-up of 6.26 years. First CVD event was defined as the initial occurrence of coronary heart disease (CHD), stroke, or heart failure (HF), and CVM was defined as the presence of two or more of these conditions. Multi-state models were used to assess the association between the TyG index and transitions across health states, including disease-specific pathways.

**Results:**

During a median follow-up of 6.26 years, 1,182 (3.79%) participants developed first CVD event, 671 (2.15%) developed CVM. TyG index played crucial but different roles in all transitions from healthy to first CVD event, to CVM (HR = 1.162, 95% CI: 1.074, 1.257; HR = 1.148, 95% CI: 1.010, 1.305). When we further divided first CVD events into CHD, Stroke, and HF, we found that TyG index played different roles in disease-speciﬁc transitions even within the same transition stage (HR = 1.145, 95% CI: 1.029, 1.273; HR = 1.228, 95% CI: 1.085, 1.391; HR = 1.213, 95% CI: 1.057, 1.391).

**Conclusions:**

The TyG index is an independent predictor of dynamic progression toward cardiovascular multimorbidity, showing significant associations with atherosclerosis-driven conditions such as CHD and stroke but not with heart failure. These findings support its potential utility in risk stratification and preventive strategies across the cardiovascular disease continuum.

## 1 Introduction

According to the Global Burden of Disease (GBD) 2023 report, cardiovascular diseases (CVDs) were the leading cause of disability-adjusted life-years (DALYs) and deaths worldwide, accounting for approximately 19.2 million fatalities (1, 2). Cardiovascular multimorbidity (CVM) is the co-occurrence of multiple cardiovascular disease subtypes (CVDs) in one person (3) and they share pathophysiological mechanisms that could be targeted for multimorbidity prevention (3–6).

In recent years, with the continuous advancement of medical technology and the aging of the population structure, the number of individuals with CVM has also been increasing significantly (7–9). More than 50% of CVD patients suffer from at least one other disease (9), multimorbidity is associated with a decrease in quality of life, an increase in mortality rate, polypharmacy, a higher incidence of adverse drug events, and extensive use of unplanned healthcare services (4, 10, 11), placing a significant burden on individuals, families, and the healthcare system. Among these, coronary heart disease(CHD), Stroke, and heart failure (HF) as the most common types of cardiovascular diseases, often interact with each other and form a vicious cycle, which is one of the important reasons for the high mortality rate in the cardiovascular and cerebrovascular systems (1–3). Considering the availability of existing data and the importance of these three diseases, In this study, CVM is specifically defined as the co-occurrence of any two or three of the following cardiovascular diseases: CHD, Stroke and HF. This definition focuses solely on the coexistence of cardiovascular conditions, excluding other non-cardiovascular and specifically metabolic diseases. The World Health Organization has unequivocally stated that the majority of cardiovascular diseases can be prevented through the management of risk factors. Therefore, identifying and managing the comorbidity of these three diseases is of great significance for reducing the incidence and mortality of cardiovascular diseases and improving the quality of life of patients.

The triglyceride-glucose (TyG) index serves as a biochemical indicator of insulin resistance (IR) in 2008 (12). It can be determined by the formula ln[fasting triglycerides (mg/dL) × fasting blood glucose (mg/dL)/2] (12). In the subsequent decade and a half, the TyG index has been consistently validated by numerous studies as an independent risk factor for various CVD, emerging as a potentially valuable tool for risk stratification and outcome prediction in CVD (13–15). However, these studies have only focused on the association of the TyG index with individual cardiovascular diseases such as Stroke (16, 17), CHD (18) and HF (19, 20). And, the research on the association between TyG and CVM is rather scarce. Current studies that do exist have primarily considered CVM as a direct outcome, identifying a correlation between a high TyG index and an increased risk of CVM events. However, these studies have not adequately taken into account the dynamic progression characteristics inherent in the development of CVD to CVM.

CVD, is inherently a series of dynamic and progressive disorders, involving an array of biological processes and factors during its developmental trajectory, and the evolution of its comorbidities is equally a long-term process (21). The rationale for investigating the TyG index in the context of CVM dynamics is strong. Insulin resistance (IR) is a shared pathophysiological mechanism that promotes the development and progression of multiple cardiovascular conditions, including atherosclerosis, hypertension, and endothelial dysfunction (12). Since the TyG index is a well-established surrogate marker of IR, it represents a plausible biological link that could drive the progression from one CVD to the accumulation of multiple conditions. Therefore, the TyG index is not merely a risk marker for individual diseases but is ideally positioned to study the dynamic process of multimorbidity accumulation.

It has been evidenced that the risk of mortality in patients afflicted with CVM is subject to temporal variation, with those experiencing incident CVM harboring a risk of subsequent mortality nearly three times elevated compared to those devoid of CVM (21), In addition, the risk change pattern in different CVD states will also change. Patients with ischemic heart disease and Stroke face a higher risk of death in the first year of onset. With the increase of disease years, Stroke patients face a higher risk of death in the long term, while the risk of death in patients with ischemic heart disease continues to decrease (21). Focusing on the dynamic progression from CVD to CVM can help to better understand the long-term impact of the disease, develop prevention strategies, optimize patient management and treatment, and improve patient outcomes. It can be concluded that the inclusion of TyG index in the multi-dynamic model for study is of great significance to further explore whether TYG plays a role in the evolution of CVM.

Therefore, we investigate the differential roles of the TyG index at various stages of cardiovascular disease progression, this study employed a multi-state model to test its stage-specific associations with transitions from CVD-free to first CVD event and (CVM) in the Shanghai Suburban Adult Cohort.

## 2 Methods

### 2.1 Data source and study population

Participants were recruited from the Shanghai Suburban Adult Cohort and Biobank (SSACB)-Songjiang Natural Adult Cohort—a prospective adult-based cohort established by the School of Public Health, Fudan University, under Shanghai's “Summit Discipline Construction Program” (22, 23). Using a multi-stage stratified cluster sampling method, participants were selected from four communities in Songjiang District (Maogang Town, Sheshan Town, Xinqiao Town, and Zhongshan Sub-district). Specifically, one-third of the neighborhood committees (or villages) were randomly sampled from each community, and a total of 36,404 residents aged 20–74 years were enrolled—including those with Shanghai household registration or having resided in Shanghai for at least five years. Baseline surveys were conducted in two batches, in April 2016 and April 2017, respectively. Follow-up data (encompassing physical examination indicators and chronic disease incidence) were collected until December 2023, thereby forming a longitudinal monitoring cohort. The study design and survey methods were described before (22).The study protocol was approved by the Medical Research Ethics Committee (ethical no: IRB#2016-04-0586-S).

We excluded participants who reported history of CHD, Stroke, HF or cancer at baseline. Participants with missing data for TyG were also excluded, leaving 31,152 participants in the present analysis.

### 2.2 Research methods

Questionnaire survey: a uniformly designed epidemiological questionnaire was used, which was asked and filled out face-to-face by trained and qualified investigators at the community health service centers where the survey subjects were located. The content of the survey includes demographics information, previous family history of disease, lifestyle and behavior. Physical examination and biochemical tests are conducted centrally by the investigator using standardized methods, all survey respondents were required to measure height, weight and blood pressure. Height was measured using a metal column stadiometer (accurate to 0.1 cm) and weight was measured using an electronic weighing scale (accurate to 0.1 kg). Body mass index (BMI) was further calculated based on height and weight. When measuring blood pressure, the subjects were asked to take a sitting position after resting for 5 min, measure the blood pressure of the brachial artery of the right arm in a quiet state, and record the blood pressure value, taking the average of three measurements (blood pressure accurate to 1mmHg, 0.133 kPa). Fasting venous blood was drawn from all study subjects and blood samples were stored and transported in a freezer. TG level was measured by enzyme colorimetric method; fasting blood glucose was measured by hexokinase method, and all biochemical tests were done in Dian Medical Laboratory Center.

### 2.3 Definitions

The baseline TyG index was utilized as the primary exposure in this study. TyG index = ln[TG(mg/dL) × FPG(mg/dL)/2] (12). The two primary outcomes of this study were the first major cardiovascular disease (CVD) event and the subsequent development of cardiovascular multimorbidity (CVM).The diagnosis of cardiovascular disease was made by clinicians according to the International Classification of Diseases, 10th Revision (ICD-10), and records were reviewed by preventive care physicians. In this study, CVM refers to Cardiovascular Multimorbidity, defined as the occurrence of two or more distinct cardiovascular diseases (including coronary heart disease, stroke, and heart failure) in the same individual.

Smoking was defined as current smoker or nonsmoker. Smoking ≥1 cigarette per day for ≥ 6 months was defined as current smoking. Alcohol consumption was defined as current drinker or non-drinker. Education is defined as college/university level or above; high school/middle school; elementary school and below, and is defined as high, middle, or low education, respectively. Adult Sleep duration is categorized into <7 h and ≥7 h, according to the the recommendations of the American Academy of Sleep Medicine and the Sleep Research Society (24). DM was defined as follows: FBG>7 mmol/L, and/or an HbA1c level ≥6.5%, and/or a self-reported/physician diagnosis diabetes mellitus (25). According to the Chinese Guidelines for the Prevention and Treatment of Hypertension (2024 Revision). Hypertension was diagnosed based on a physician diagnosis hypertension and/or rasystolic blood pressure/diastolic blood pressure (SBP/DBP) ≥140/90 mmHg (26).

### 2.4 Statistical analysis

Statistical analysis of the data was conducted using R version 4.3.3.Participants were grouped according to their cardiovascular disease status: those with no history of cardiovascular disease (CVD-free), those with a first occurrence of cardiovascular disease (first CVD event), and those with CVM. Categorical variables were assessed for distribution differences across these groups using the chi-square test. For the continuous variable TyG index, which exhibited a skewed distribution, it was described using the median and interquartile range [M(IQR)]. The Mann–Whitney *U*-test was employed to evaluate differences in the TyG index among the different groups. To address missing covariate data, we performed multiple imputation using the R package mice (5 imputations). Parameter estimates from the multi-state models fitted on each imputed dataset were pooled using Rubin's rules.

**Figure 1:**
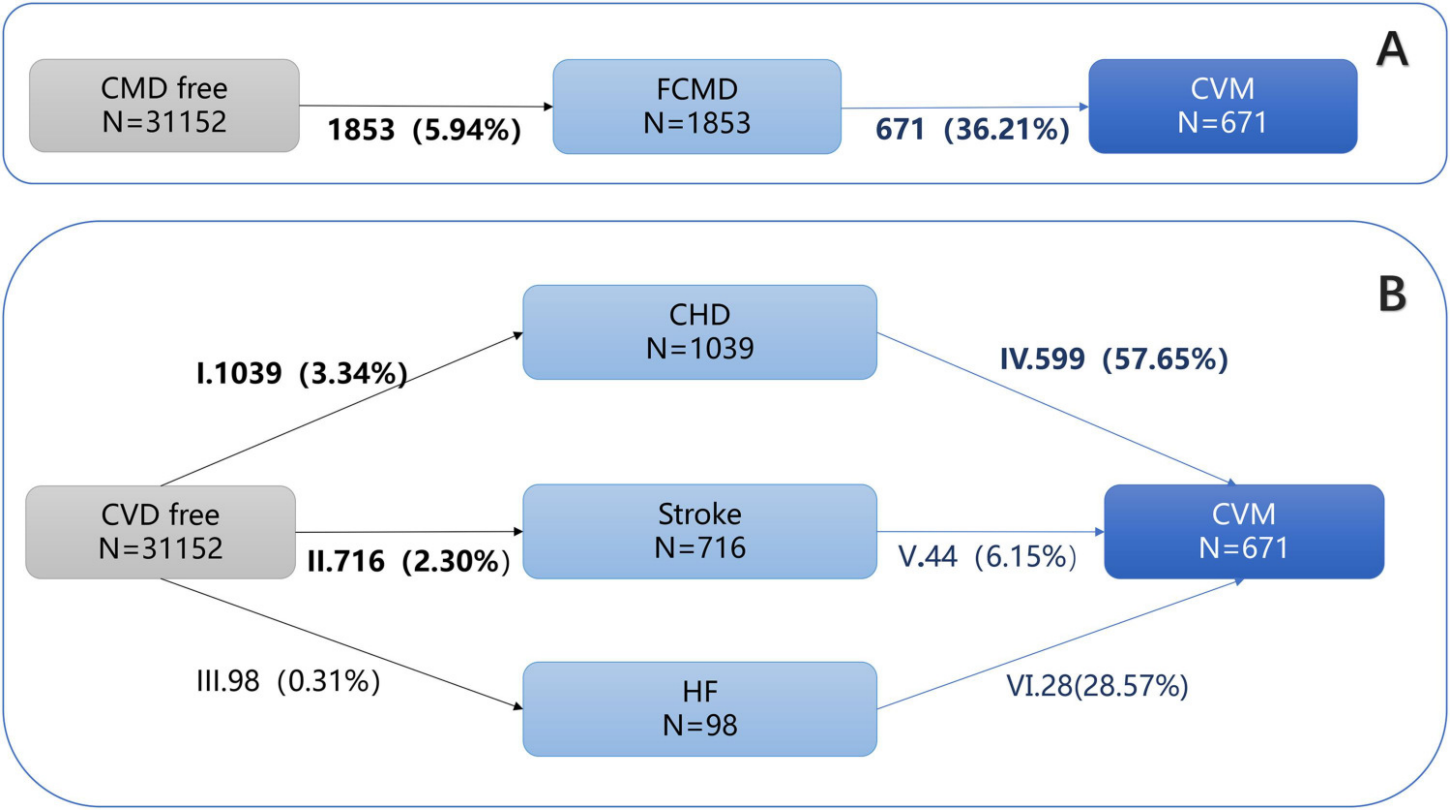
Numbers (percentages) of participants in multistate models. **(A)** Overall multistate model; **(B)** Specific multistate model. CVD, Cardiovascular Disease; CHD, Coronary Heart disease; CVM, Cardiovascular Multimorbidity. Transition I: from CVD-free to CHD; Transition Ⅱ: from CVD-free to Stroke; Transition Ⅲ: from CVD-free to HF; Transition Ⅳ: from CHD to CVM; Transition Ⅴ: from Stroke to CVM; Transition Ⅵ: from HF to CVM.

The multi-state model employed in this study divided the occurrence and development of CVD into two transition processes: from CVD-free to first CVD event and from first CVD event to CVM (Figure 1A). Under consideration of competing risks, this approach allows for a more in-depth understanding of the process of CVD occurrence and development (27). Mutually exclusive endpoints were modeled as distinct absorbing states and individuals experiencing a competing event were transitioned to that state rather than censored. We estimated transition-specific hazards for all allowed transitions, thereby properly accounting for competing non-death events. The impact of TyG was estimated in both processes. To further investigate the effects of TyG on different types of CVD, this study subdivided first CVD event into three diseases (CHD, Stroke, HF), resulting in six transition processes, as shown in Figure 1B. The impact of TyG was estimated in all six transition processes.

Sensitivity analysis was conducted to evaluate the robustness of the associations. To examine their independence from systemic inflammation, we repeated the multi-state models after adjusting for white blood cell count (WBC) and low-density lipoprotein cholesterol (LDL-C).

## 3 Results

### 3.1 Baseline characteristics

Table 1 presents the baseline characteristics of the 31,152 participants free of cardiovascular disease (CVD) at enrollment, who were followed until 2023. Over a median follow-up of 6.52 years (interquartile range: 6.08–6.83 years), 1,182 (3.79%) participants experienced at least one incident CVD event. Among these, 671 (2.15%) developed cardiovascular mortality (CVM). The study population was predominantly aged 45–74 years, with a male predominance (59.40%). Significant differences (*p* < 0.05) were observed in the distribution of age, sex, body mass index (BMI), marital status, educational level, sleep duration, family history of CVD, alcohol consumption, smoking status, hypertension, and diabetes mellitus across groups defined by incident CVD, CVM, or remaining CVD-free. Participants who developed CVD during follow-up were generally older, had lower educational attainment, higher rates of smoking and alcohol use, greater prevalence of hypertension and diabetes, and higher BMI compared to those who remained free of cardiovascular events (*p* < 0.05).

**Table 1:** Baseline characteristics of the study participants (*n* = 31,152).

Variables	Overall	CVD free	First CVD event	CVM	*P* value
	(*n* = 31,152)	(*n* = 29,299)	(*n* = 1,182)	(*n* = 671)	
TyG, median (IQR)					
	8.56 (8.20, 8.96)	8.55 (8.19, 8.95)	8.68 (8.29, 9.12)	8.69 (8.27, 9.13)	<0.001
Age (years), *n* (%)					
20–44	4,966 (15.94)	4,921 (16.80)	34 (2.88)	11 (1.64)	<0.001
45–74	26,186 (84.06)	24,378 (83.20)	1,148 (97.12)	660 (98.36)	
Sex, *n* (%)					
Female	12,649 (40.60)	11,694 (39.91)	621 (52.54)	334 (49.78)	<0.001
Male	18,503 (59.40)	17,605 (60.09)	561 (47.46)	33 (50.22)	
BMI, *n* (%), kg/m^2^					
<25 kg/m^2^	18,794 (60.33)	17,842 (60.90)	610 (51.61)	342 (50.97)	<0.001
≥25 kg/m^2^	12,358 (39.67)	11,457 (39.10)	572 (48.39)	329 (49.03)	
Marital status, *n* (%)					
Married	28,993 (93.07)	27,287 (93.13)	1,102 (93.23)	604 (90.01)	0.007
Unmarried	2,159 (6.93)	2,012 (6.87)	80 (6.77)	67 (9.99)	
Education, *n* (%)					
Low	13,745 (44.12)	12,593 (42.98)	70 (59.90)	444 (66.17)	<0.001
Middle	1,529 (49.10)	14,611 (49.87)	459 (38.83)	225 (33.53)	
High	2,112 (6.78)	2,095 (7.15)	15 (1.27)	2 (0.30)	
Sleeptime, *n* (%)					
<7 h	13,420 (43.08)	12,563 (42.88)	535 (45.26)	322 (47.99)	0.009
≥7 h	17,732 (56.92)	16,736 (57.12)	647 (54.74)	349 (52.01)	
Family history of CVD, *n* (%)					
No	27,701 (88.92)	26,051 (88.91)	1,047 (88.58)	603 (89.87)	0.687
Yes	3,451 (11.08)	3,248 (11.09)	135 (11.42)	68 (10.13)	
Smoking statues, *n* (%)					
No	23,725 (76.16)	22,468 (76.69)	802 (67.85)	455 (67.81)	<0.001
Yes	7,427 (23.84)	6,831 (23.31)	380 (32.15)	216 (32.19)	
Drinking statues, *n* (%)					
No	26,899 (86.35)	25,392 (86.67)	960 (81.22)	547 (81.52)	<0.001
Yes	4,253 (13.65)	3,907 (13.33)	222 (18.78)	124 (18.48)	
Diabetes Mellitus, *n* (%)					
No	29,392 (94.35)	27,793 (94.86)	1,599 (86.29)	571 (85.10)	<0.001
Yes	1,760 (5.65)	1,506 (5.14)	254 (13.71)	100 (14.90)	
Hypertension, *n* (%)					
No	21,648 (69.49)	20,715 (70.70)	933(50.35)	301(44.86)	<0.001
Yes	9,504 (30.51)	8,584(29.30)	920(49.65)	370(55.14)	

Data are presented as median(quantile 1, quantile 3) or number (%), as appropriate.

### 3.2 Multistate analyses

Main findings: of the 31,152 participants, 1,853 (5.94%) developed a first CVD event, and 36.21% of these patients progressed to CVM. **A multi-state model demonstrated that the TyG index was a significant risk factor for both transitions in the CVD continuum:** from a CVD-free state tofirst CVD event (HR = 1.162, 95% CI: 1.074–1.257) and from first CVD event to CVM (HR = 1.148, 95% CI: 1.010–1.305) (Table 2).

**Table 2:** Associations between TyG and different transition stages.

Models	CVD-free → first CVD event	P	First CVD event → CVM	P
	HR (95% CI)		HR (95% CI)	
Model 0	1.446 (1.347, 1.552)	<0.001	1.131 (1.003, 1.274)	0.044
Model 1	1.304 (1.210, 1.406)	<0.001	1.156 (1.023, 1.306)	0.020
Model 2	1.304 (1.209, 1.406)	<0.001	1.166 (1.031, 1.317)	0.014
Model 3	1.162 (1.074, 1.257)	<0.001	1.148 (1.010, 1.305)	0.035

Model 0: crude model.

Model 1: adjusted for age, gender, BMI, Marital Status, education.

Model 2: adjusted for age, gender, BMI, Marital Status, education, Smoking statues, drinking statues.

Model 3: adjusted for age, gender, BMI, Marital Status, education, Smoking statues, drinking statues, family history of cvd, Diabetes Mellitus, Hypertension.

Notably, the magnitude of the association (Hazard Ratio) for the first transition (CVD-free to first CVD event) was consistently stronger than that for the second transition first CVD event to CVM) across all stages of adjustment (Figure 1A). This suggests that the effect of the TyG index might be more potent in the pre-disease (healthy) population.

### 3.3 Associations between the TyG index and individual CVD transitions

Coronary heart disease, Stroke, and heart failure occurred in 1,039 (3.34%), 716 (2.30%), and 98(0.31%) participants, respectively, among those without cardiovascular disease at baseline. Subdividing the first CVD event stage into three different cardiac diseases (CHD, STOKE, HF) constructed six transition stages: (i) from CVD-free to CHD; (ii) from CVD-free to Stroke; (iii) from CVD-free to HF; (iv)from CHD to CVM; (v): from Stroke to CVM; (Ⅵ): from HF to CVM (Table 3, Figure 1B).

**Table 3:** Hazard ratios (95% CIs) for each transition in transition pattern B by TyG among 31,152 participants.

Models	Six transition stages	HR (95% CI)	*P* value
Transition 1	From CVD-free to CHD	1.145 (1.029, 1.273)	0.013
Transition 2	from CVD-free to Stroke	1.228 (1.085, 1.391)	0.001
Transition 3	from CVD-free to HF	0.893 (0.628, 1.269)	0.529
Transition 4	from CHD to CVM	1.213 (1.057, 1.391)	0.006
Transition 5	from Stroke to CVM	1.042 (0.614, 1.767)	0.879
Transition 6	from HF to CVM	1.056 (0.422, 2.646)	0.907

The model was adjusted for age, gender, BMI, Marital Status, education, Smoking statues, drinking statues, family history of cvd, Diabetes Mellitus, Hypertension.

The result showed that the TyG index had differential impacts on different types of cardiovascular diseases within the same phase: TyG index is a risk factor that promotes the development of CHD, and the effect of TyG index on Stroke is significant at the stage from health to first Stroke diagnosis.

### 3.4 Sensitivity analyses

Considering that the occurrence and development of CVD involve multiple stages, each with different mechanisms interacting with one another (9). A high white blood cell count (WBC) may indicate an inflammatory state, and low-density lipoprotein cholesterol (LDL-C) is one of the major risk factors for cardiovascular disease. Abnormalities in these biomarkers may signal an increased risk of cardiovascular disease (28). Based on these considerations, we continued to adjust the levels of WBC and LDL-C in the sensitivity analysis, The results showed that TyG index was still significantly associated with the risk of cardiovascular disease from CVD-free to first CVD event, and from first CVD event to CVM (*p* < 0.05) (Table 4).

**Table 4:** Sensitivity Analysis of TyG Index and Cardiovascular Disease multimorbidity.

Models	CVD-free → first CVD event	*P*	First CVD event → c	*P*
	HR (95% CI)		HR (95% CI)	
Model 1	1.140 (1.052, 1.235)	0.001	1.151 (1.010, 1.313)	0.035
Model 2	1.173 (1.082, 1.272)	<0.001	1.150 (1.012, 1.307)	0.032

Model 1: adjusted for age, gender, BMI, Marital Status, education, Smoking statues, drinking statues, family history of cvd, Diabetes Mellitus, Hypertension, WBC.

Model 2: adjusted for age, gender, BMI, Marital Status, education, Smoking statues, drinking statues, family history of cvd, Diabetes Mellitus, Hypertension, LDL-C.

## 4 Discussion

This study employed a multi-state model in a cohort of 31,152 Chinese adults to characterize the TyG index's role in the dynamic clinical course from health to first CVD (first CVD event) and then to CVM. Our results demonstrate that a higher TyG index significantly accelerates this progression. Moreover, for each unit increase in the TyG index, the risks of transitioning from a CVD-free state to first CVD event and from first CVD event to CVM increased by 1.162-fold (HR = 1.162, 95% CI: 1.074–1.257) and 1.148-fold (HR = 1.148, 95% CI: 1.010–1.305), respectively. In line with existing literature, our study confirms that a higher TyG index is associated with an increased CVD risk (29–31), thereby strengthening the evidence base for its predictive validity.

A key contribution of this study is its dynamic perspective, which moves beyond the static, single-endpoint focus of prior research. Using a multi-state model, we demonstrate that the TyG index not only accelerates the transition from health to first-onset cardiovascular disease (CVD) but also differentially influences specific cardiovascular outcomes across the disease continuum. A noteworthy finding, which persisted after adjustment for key confounders, was that the TyG index was a strong risk factor for incident coronary heart disease (CHD) and significantly associated with stroke onset, yet showed no significant association with progression to heart failure (HF). This heterogeneity underscores the value of a dynamic modeling approach for refined risk stratification and suggests distinct pathophysiological pathways link insulin resistance to different CVD manifestations.

The robust association with CHD and stroke is biologically plausible, as atherosclerosis is the cornerstone pathophysiological process for both. The TyG index, as a marker of insulin resistance (IR), promotes a cascade of pro-atherogenic mechanisms including endothelial dysfunction, inflammation, oxidative stress, and thrombosis (32). The null finding regarding HF, however, warrants a deeper mechanistic interpretation. This is unlikely to be a true null effect but rather reflects the distinct pathophysiology of HF subtypes. Contemporary evidence firmly establishes that IR is a central driver of heart failure with preserved ejection fraction (HFpEF), which is often intertwined with obesity and metabolic syndrome, whereas its link to heart failure with reduced ejection fraction (HFrEF) is weaker (20, 33, 34). Our study, lacking relatively number of HF cases, likely masked a specific association with HFpEF. Furthermore, the path from IR to CVD is multifaceted. IR can induce glucolipotoxicity and promote sympathetic overactivity, potentially leading to vascular dysfunction and myocardial injury (35). The inconsistent results across studies regarding HF (20) may stem from variations in the prevalence of HFpEF within different cohorts, alongside differences in sample size and study design.

At present, the mechanism by which TyG index plays a role in multimorbidity is not clear, but the reasons may be as follows: first, elevated TyG index increases the risk of CVD by being associated with multiple mechanisms such as insulin resistance, endothelial cell damage, inflammation, oxidative stress, thrombosis and so on (36, 37). These mechanisms work together to promote the development of atherosclerosis and increase the risk of cardiovascular events, However, in the pathogenesis of CVD, the accumulation of body fat increases insulin resistance, dyslipidemia, and cytokines, which further increase the risk of CVM (37). Secondly, insulin can further cause lipid hyaluronic acid degeneration by enhancing sympathetic nerve activity or acting as a growth factor, and lipophosphate deposition can block small arteries, leading to the occurrence of CVD (35); IR can induce glucose metabolism disorders and lipotoxicity, and may also lead to the inactivation of nitric oxide and the excessive production of reactive oxygen species, resulting in the inflammation and dysfunction of vascular endothelium, resulting in the occurrence of a variety of chronic diseases (35).

## 5 Conclusion and limitations

In conclusion, this study provides novel evidence suggesting that the TyG index differentially influences dynamic CVD transitions. Our findings highlight the potential of the TyG index as a low-cost predictive tool for risk stratification across much of the CVD spectrum, particularly for atherosclerosis-driven conditions.

However, several limitations of this study must be considered when interpreting the results. First, our operational definition of CVM was necessarily restricted to the co-occurrence of CHD, stroke, and heart failure. While this allowed for clear definition and analysis, it may not fully capture the complexity of real-world multimorbidity, as other prevalent cardiometabolic conditions (e.g., peripheral artery disease, atrial fibrillation, chronic kidney disease) were not included. This narrow definition could lead to an underestimation of the true multimorbidity burden associated with the TyG index. Future studies employing broader, more inclusive definitions of CVM are warranted.

Second, despite adjusting for major clinical covariates, residual confounding remains possible due to the lack of data on several key variables. Specifically, our analysis lacked information on medication use (e.g., statins, antihypertensives), detailed lifestyle factors (e.g., diet, physical activity), specific inflammatory biomarkers, and most critically, mortality records. These unmeasured factors could influence both the TyG index and CVD risk, potentially biasing the observed associations.

Third, our study population was drawn from a single district in Shanghai. The generalizability of our findings to populations with different genetic backgrounds, healthcare access, dietary habits, and environmental exposures across China and globally requires validation through future multi-center studies. Additionally, mortality data were unavailable in this study. Therefore, we have not considered the competing risk of death.

Finally, the use of a single baseline TyG measurement means we could not account for its longitudinal variation. Therefore, our analysis cannot establish causality and might underestimate the true association, as changes over time due to treatment or lifestyle likely influence risk. Future research with repeated measures is needed to provide a more dynamic and accurate assessment of this relationship.

Notwithstanding these limitations, our study offers valuable insights. It underscores the potential of the TyG index as a practical tool for large-scale risk screening and sets the stage for more personalized prevention strategies and rigorous interventional trials.

## Data Availability

The original contributions presented in the study are included in the article/Supplementary Material, further inquiries can be directed to the corresponding authors.
