# Supplementary material for: Triglyceride-glucose index as a predictor of cardiovascular multimorbidity: a prospective cohort study
Source: Front Cardiovasc Med. 2025 Nov 14;12:1647444. doi: 10.3389/fcvm.2025.1647444 (PMC12660249; doi:10.3389/fcvm.2025.1647444)
Supplement: Supplementary file 1 [file Datasheet1.pdf]

Supplemental Files

Corresponding to article Table 2

Supplement table1:**Associations between tyg and different transition stage**

models		CVD-free→FCVD<br>HR (95%CI)	P	FCVD→MCVD<br>HR (95%CI)	P
model0	**TYG**	**1.446 (1.347,1.552)**	**<0.001**	**1.131(1.003,1.274)**	**0.044**
model1	**TYG**	**1.304 (1.210,1.406)**	**<0.001**	**1.156 (1.023,1.306)**	**0.020**
	age45	4.747 (3.501,6.438)	<0.001	1.627 (0.885,2.992)	0.117
	sex	0.583 (0.531,0.640)	<0.001	0.964 (0.825,1.126)	0.643
	BMI25	1.172 (1.067,1.288)	<0.001	1.105 (0.947,1.290)	0.205
	education	0.545 (0.496,0.598)	<0.001	0.723 (0.613,0.853)	<0.001
	MaritalStatus	1.253 (1.058,1.485)	0.009	1.429 (1.106,1.846)	0.006
model2	**TYG**	**1.304 (1.209,1.406)**	**<0.001**	**1.166 (1.031,1.317)**	**0.014**
	age45	4.733 (3.489,6.419)	<0.001	1.710 (0.926,3.157)	0.086
	BMI25	1.176 (1.070,1.292)	<0.001	1.101 (0.943,1.286)	0.222
	sex	0.602 (0.530,0.683)	<0.001	0.863 (0.692,1.076)	0.189
	education	0.544 (0.495,0.598)	<0.001	0.714 (0.605,0.844)	<0.001
	MaritalStatus	1.254 (1.059,1.487)	0.009	1.447 (1.119,1.870)	0.005
	SMOKE	0.928 (0.812,1.060)	0.273	1.072 (0.853,1.348)	0.550
	drink	1.040 (0.910,1.190)	0.564	1.180 (0.941,1.482)	0.152
model3	**TYG**	**1.162 (1.074,1.257)**	**<0.001**	**1.148 (1.010,1.305)**	**0.035**
	age45	4.229 (3.113,5.744)	<0.001	1.695 (0.916,3.136)	0.093
	sex	0.624 (0.549,0.708)	<0.001	0.868 (0.696,1.083)	0.210
	BMI25	1.076 (0.977,1.184)	0.137	1.059 (0.905,1.240)	0.473
	education	0.580 (0.527,0.638)	<0.001	0.737 (0.623,0.873)	<0.001
	MaritalStatus	1.239 (1.046,1.469)	0.013	1.442 (1.116,1.864)	0.005
	SMOKE	0.898 (0.786,1.026)	0.115	1.072 (0.853,1.349)	0.550
	drink	1.071 (0.936,1.225)	0.318	1.203 (0.957,1.513)	0.112
	familyhistoryofcvd	0.991 (0.856,1.147)	0.906	0.806 (0.626,1.038)	0.094
	Diabetes Mellitus	1.592 (1.371,1.849)	<0.001	1.044 (0.818,1.332)	0.729
	Hypertension	1.525 (1.376,1.690)	<0.001	1.226 (1.036,1.452)	0.018

Model0: crude model

Model 1: adjusted for age,gender,BMI,MaritalStatus,education

Model 2: adjusted forage,gender,BMI,MaritalStatu,education,SMOKing statues,drinking statues,

Model 3:adjusted forage,gender,BMI,MaritalStatu,education,,SMOKing statues,drinking statues,familyhistoryofcvd,Diabetes Mellitus,Hypertension

Corresponding to article Table 3

Supplement Table2:Hazard ratios (95% CIs) for each transition in transition pattern B by tyg among 31152 participants

models	HR (95%CI)	*P*	HR (95%CI)	*P*

Transition 1				Transition 2		
	**TYG**	**1.145 (1.029,1.273)**	**0.013**		**1.228 (1.085,1.391)**	**0.001**
	age45	7.498 (4.378,12.842)	<0.001		3.694 (2.327,5.865)	<0.001
	sex	0.655 (0.552,0.777)	<0.001		0.585 (0.477,0.716)	<0.001
	BMI25	1.103 (0.970,1.253)	<0.001		1.031 (0.883,1.203)	0.701
	education	0.569 (0.501,0.647)	<0.001		0.579 (0.497,0.675)	<0.001
	MaritalStatus	1.141 (0.903,1.441)	0.270		1.209 (0.916,1.595)	<0.001
	SMOKE	0.894 (0.746,1.072)	0.226		0.927 (0.751,1.143)	0.477
	drink	1.168 (0.971,1.404)	0.099		0.939 (0.762,1.157)	0.553
	familyhistoryofcvd	1.059 (0.876,1.280)	0.553		0.939 (0.737,1.195)	0.607
	DM	1.316 (1.072,1.616)	0.009		1.854 (1.463,2.350)	<0.001
	hypertension	1.716 (1.500,1.964)	<0.001		1.330 (1.124,1.574)	<0.001
Transition 3				Transition 4		
	TYG	0.893 (0.628,1.269)	0.529		**1.213 (1.057,1.391)**	**0.006**
	age45	1.027 (0.497,2.120)	0.942		0.824 (0.433,1.567)	0.554
	sex	0.596 (0.344,1.033)	0.065		0.857 (0.677,1.085)	0.200
	BMI25	1.129 (0.739,1.726)	0.575		0.993 (0.839,1.176)	0.937
	education	0.690 (0.464,1.027)	0.068		0.725 (0.606,0.866)	<0.001
	MaritalStatus	2.586 (1.483,4.508)	<0.001		1.540 (1.168,2.032)	0.002
	SMOKE	0.737 (0.416,1.305)	0.295		1.182 (0.920,1.517)	0.190
	drink	1.202 (0.667,2.165)	0.541		1.173 (0.915,1.504)	0.208
	familyhistoryofcvd	0.651 (0.301,1.407)	0.275		0.766 (0.587,0.999)	0.049
	Diabetes Mellitus	3.932 (2.099,7.365)	<0.001		1.368 (1.044,1.792)	0.023
	hypertension	1.036 (0.624,1.718)	0.892		0.995 (0.833,1.189)	0.958
Transition 5				Transition 6		
	TYG	1.042 (0.614,1.767)	0.879		1.056 (0.422,2.646)	0.907
	age45	0.809 (0.097,6.781)	0.845		1.170 (0.000,Inf)	0.998
	sex	0.542 (0.238,1.234)	0.144		0.369 (0.125,1.091)	0.0714
	BMI25	0.600 (0.319,1.130)	0.114		1.093 (0.416,2.876)	0.856
	education	0.756 (0.387,1.476)	0.413		0.256 (0.091,0.718)	0.010
	MaritalStatus	1.625 (0.674,3.920)	0.280		0.863 (0.229,3.251)	0.828
	SMOKE	1.081 (0.478,2.442)	0.852		0.401 (0.142,1.133)	0.085
	drink	1.486 (0.642,3.444)	0.355		1.030 (0.328,3.231)	0.960
	familyhistoryofcvd	0.509 (0.155,1.671)	0.265		1.388 (0.326,5.911)	0.657
	Diabetes Mellitus	0.803 (0.344,1.871)	0.610		4.000 (1.025,15.613)	0.046
	hypertension	3.583 (1.728,7.430)	<0.001		0.984 (0.330,2.938)	0.978

The model was adjusted for age,gender,BMI,MaritalStatu,education,,SMOKing statues,drinking statues,familyhistoryofcvd,Diabetes

Sensitivity analyses

Considering that the occurrence and development of CVD involve multiple stages, each with different mechanisms interacting with one another[9]. A high white blood cell count (WBC) may indicate an inflammatory state, and low-density lipoprotein cholesterol (LDL-C) is one of the major risk factors for cardiovascular disease. Abnormalities in these biomarkers may signal an increased risk of cardiovascular disease[27].

Supplement 3:Table 4: Sensitivity Analysis of TYG Index and Cardiovascular Disease multimorbidity

models		CVD-free→FCVD HR (95%CI)	*P*	FCVD→MCVD HR (95%CI)	*P*
Model1	**TYG**	**1.140 (1.052,1.235)**	**0.001**	**1.151 (1.010,1.313)**	**0.035**
	age45	4.301 (3.165,5.844)	<0.001	1.690 (0.913,3.129)	0.095
	sex	0.633 (0.557,0.719)	<0.001	0.867 (0.694,1.082)	0.206
	BMI25	1.067 (0.969,1.175)	0.185	1.061 (0.906,1.243)	0.463
	education	0.579 (0.526,0.636)	<0.001	0.737 (0.623,0.873)	<0.001
	MaritalStatus	1.238 (1.044,1.467)	0.014	1.442 (1.116,1.864)	0.005
	SMOKE	0.912 (0.798,1.042)	0.177	1.071 (0.851,1.347)	0.559
	drink	1.061 (0.927,1.213)	0.390	1.204 (0.958,1.514)	0.111
	familyhistoryofcvd	0.991 (0.856,1.147)	0.899	0.807 (0.626,1.039)	0.096
	Diabetes Mellitus	1.577 (1.358,1.832)	<0.001	1.046 (0.819,1.335)	0.720
	hypertension	1.515 (1.367,1.679)	<0.001	1.228 (1.036,1.454)	0.018
	WBC	1.043 (1.013,1.074)	0.005	0.995 (0.945,1.047)	0.843
Model2	**TYG**	**1.173 (1.082,1.272)**	**<0.001**	**1.150 (1.012,1.307)**	**0.032**
	age45	4.001 (2.945,5.437)	<0.001	1.711 (0.923,3.169)	0.088
	sex	0.599 (0.527,0.681)	<0.001	0.870 (0.697,1.085)	0.217
	BMI25	1.064 (0.967,1.172)	0.204	1.060 (0.906,1.241)	0.466
	education	0.582 (0.529,0.640)	<0.001	0.735 (0.621,0.870)	<0.001
	MaritalStatus	1.241 (1.047,1.470)	0.013	1.442 (1.116,1.864)	0.005
	SMOKE	0.898 (0.786,1.026)	0.114	1.072 (0.852,1.348)	0.553
	drink	1.076 (0.941,1.231)	0.286	1.207 (0.960,1.518)	0.107
	familyhistoryofcvd	0.988 (0.853,1.143)	0.870	0.808 (0.627,1.040)	0.098
	Diabetes Mellitus	1.619 (1.393,1.881)	<0.001	1.040 (0.815,1.328)	0.751
	hypertension	1.531 (1.382,1.697)	<0.001	1.223 (1.032,1.449)	0.020
	LDL-C	1.227 (1.164,1.293)	<0.001	0.978 (0.900,1.063)	0.603

Model 1:adjusted forage,gender,BMI,MaritalStatu,education,,SMOKing statues,drinking statues,familyhistoryofcvd,Diabetes Mellitus,Hypertension,WBC.

Model 2:adjusted forage,gender,BMI,MaritalStatu,education,,SMOKing statues,drinking statues,familyhistoryofcvd,Diabetes Mellitus,Hypertension,LDL-C.

Flow diagram of participant selection in the present analysis：

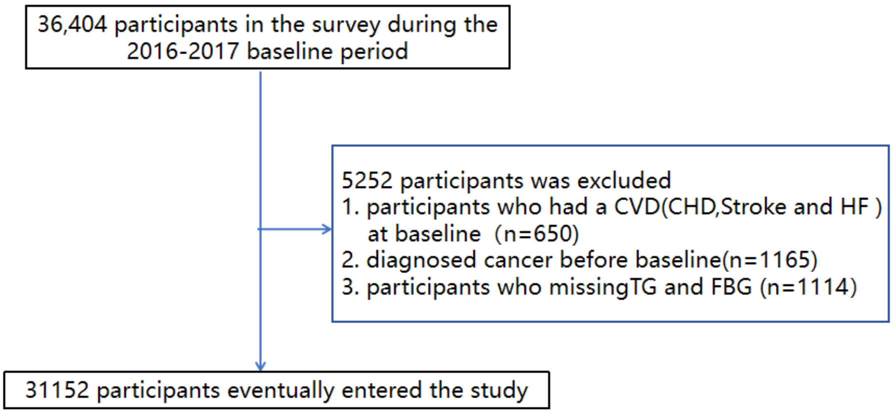

supplement picture1. Flow diagram of participant selection in the present analysis.

References

[1] Liu G, Xue Y, Liu Y, et al. Multimorbidity in cardiovascular disease and association with life satisfaction: A Chinese national cross-sectional study[J]. BMJ open, 2020, 10(12): e042950.

[2] Chinese Society of Cardiology, Chinese Society of Rehabilitation Medicine, Chinese Society of Cardiac Prevention and Rehabilitation, Chinese Society of Geriatrics and Geriatrics, et al. Chinese guidelines for the primary prevention of cardiovascular disease [J]. Chin J Cardiol, 2020, 48(12): 1000-1038.
